# Diagnostic Dilemma of Paraneoplastic Rheumatic Disorders: Case Series and Narrative Review

**DOI:** 10.7759/cureus.19993

**Published:** 2021-11-29

**Authors:** Youngmin Cho, Erik W Anderson, Sara J Guevara, Santiago J Miyara, Naomi Maria, Christine N Metz, Stefanos Zafeiropoulos, Dimitrios Giannis, Jifeng Wang, Oluseyi Abidoye, James M Mumford, Judith Aronsohn, Ernesto Molmenti, Huma Sohail

**Affiliations:** 1 Internal Medicine, Northeast Georgia Medical Center Gainesville, Gainesville, USA; 2 Rheumatology, Coney Island Hospital, Brooklyn, USA; 3 Surgery, North Shore University Hospital, Manhasset, USA; 4 Critical Care, North Shore University Hospital, Manhasset, USA; 5 Molecular Medicine, Feinstein Institutes for Medical Research, Manhasset, USA; 6 Cardiology, Serres General Hospital, Serres, GRC; 7 Family Medicine, Glen Cove Hospital, Glen Cove, USA; 8 Anesthesiology, North Shore University Hospital, Manhasset, USA; 9 Transplant Surgery, North Shore University Hospital, Manhasset, USA; 10 Rheumatology, Northeast Georgia Medical Center Gainesville, Gainesville, USA

**Keywords:** connective tissue disease, autoimmunity, malignancy, rheumatoid disorder, paraneoplastic syndromes

## Abstract

Paraneoplastic rheumatic disorder (RD) is a disorder that may present before, concurrent with, or after the diagnosis of malignancy. Paraneoplastic RDs are a clinical expression of occult cancer that is not directly related to a tumor or metastasis and manifests as rheumatoid symptoms. The RD is determined by the organ system affected by articular, muscular, cutaneous, vascular, or miscellaneous symptoms. Each case is challenging to diagnose because cancer may present with similar symptoms as a common rheumatic disorder. Of note, the majority of cases have minimal responsiveness or no responsiveness to standard rheumatoid treatment. Therefore, it is imperative to recognize and treat the underlying cancer accordingly. Herein, we present four different diagnostic dilemma cases of RD: case #1 - leukocytoclastic vasculitis and C3 glomerulopathy, case #2 - scleroderma, case #3 - Raynaud’s syndrome and possible lupus-like syndrome, and case #4 - inflammatory myositis. Institutional IRB approval was obtained for this case series. We will discuss and review the literature on each topic. In addition, we will mention a review of paraneoplastic rheumatoid arthritis. As rheumatic disease is associated with the use of immune checkpoint inhibitors (ICIs) for cancer treatment, we will briefly discuss some of the most common rheumatic presentations in the setting of these drugs. This case review aims to inform clinicians about the atypical presentation of paraneoplastic RD and to highlight the need for interdisciplinary management between rheumatologists, oncologists, and primary care practitioners.

## Introduction

A paraneoplastic rheumatic disorder (RD) is a process that refers to the expression of rheumatic disease in the context of a malignancy and is not directly caused by tumor invasion or metastasis. The most common reported paraneoplastic rheumatic syndromes are hypertrophic osteoarthropathy (HOA), seronegative polyarthritis, inflammatory myositis, paraneoplastic eosinophilic fasciitis, paraneoplastic lupus-like syndrome, recurrent polychondritis, panniculitis, erythema nodosum, and Raynaud’s syndrome [1-3]. In this case series, we will discuss four diagnostic dilemmas of the paraneoplastic syndrome: case #1 - leukocytoclastic vasculitis and C3 glomerulopathy, case #2 - scleroderma, case #3 - Raynaud’s syndrome, and case #4 - inflammatory myositis. In addition, we will mention a review of paraneoplastic rheumatoid arthritis. As rheumatic disease is associated with the use of immune checkpoint inhibitors (ICIs) for cancer treatment, we will briefly discuss some of the most common rheumatic presentations in the setting of these drugs. This review aims to describe the varied presentation of paraneoplastic disease, the existing hypotheses regarding etiology, challenges in the differential diagnosis and treatment, and the importance of early diagnosis and treatment of an occult neoplasm.

## Case presentation

Case #1: paraneoplastic leukocytoclastic vasculitis and C3 glomerulopathy

**Figure 1:**
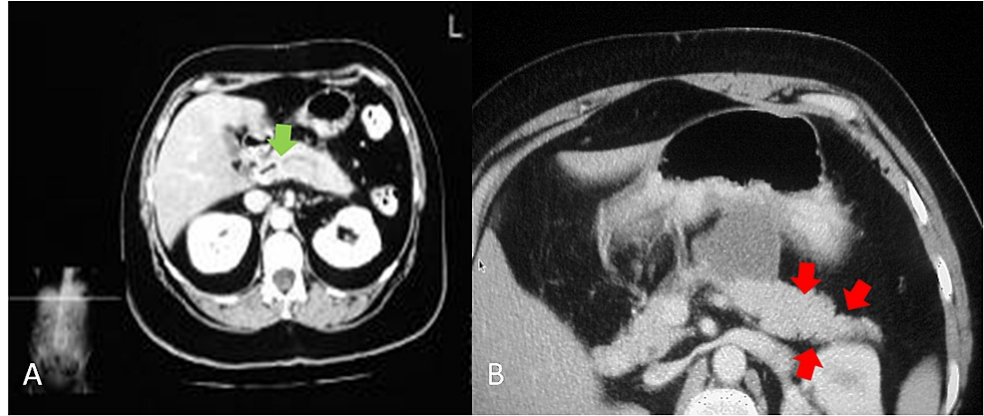
MRI of the abdomen with contrast A) diffuse slight dilatation of the main pancreatic duct with slightly prominent side branches most suggestive of chronic pancreatitis (green arrow); B) fat stranding surrounding the pancreatic body and tail (red arrows)

A 56-year-old male without a relevant past medical history was admitted for an episode of pancreatitis that evolved over two weeks and ameliorated with standard treatment. However, the following day, the patient developed two episodes of a purpuric, palpable skin rash. The first episode was diffusely distributed and the second was restricted to the lower limbs. A skin biopsy demonstrated leukocytoclastic vasculitis. Based on a worsening protein/creatinine ratio of 0.52 mg/mg (normal value: < 0.42 mg/mg) and urine analysis with dysmorphic red blood cells, the patient underwent a kidney biopsy that showed basement membrane zone deposition of C3 without immunoglobulin G (IgG) or immunoglobulin A (IgA) deposition. The tumor markers, cancer antigen 19-9 (CA 19-9), carcinoembryonic antigen (CEA), and alpha-fetoprotein (AFP), were normal. Laboratory studies showed a serum IgG4 level of 216 (normal range (NR): 8 - 150 mg/dL), amylase of 253 (NR: 30 - 110 U/L), and lipase of 88 (NR: 0 - 160 U/L). Hepatitis B and C serology and extractable nuclear antigen (ENA) 11 were negative. A serological workup revealed negative anti-neutrophil cytoplasmic antibodies (ANCAs) against proteinase 3 (PR3) and myeloperoxidase (MPO) with normal complement levels. Abdominal magnetic resonance imaging (MRI) with contrast showed diffuse, mild dilation of the main pancreatic duct with prominent side branches and stricture of the distal common bile duct, suggestive of chronic pancreatitis. Two adjacent fluid collections were observed suggesting pseudocysts on the tail (Figure 1). 

The patient was treated for cutaneous vasculitis with colchicine, steroid cream, and non-steroidal anti-inflammatory drugs (NSAIDs) without improvement. Subsequent abdominal computed tomography (CT) showed a prominent and indistinct pancreatic head with heterogeneity. A transesophageal ultrasound revealed an irregular, mass-like area identified in the pancreatic head. The mass was hypoechoic (28 mm/24 mm), and a fine-needle aspiration biopsy revealed invasive ductal adenocarcinoma.

Case #2: paraneoplastic scleroderma

**Figure 2:**
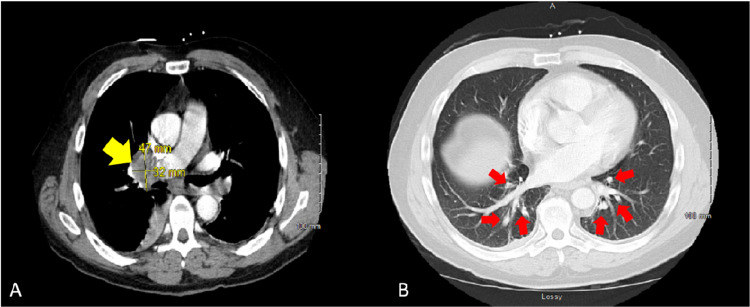
Computed tomography angiography (CTA) of the chest A) large right hilar mass measuring 47 x 32 mm (yellow arrow) in association with consolidation to the medial right lower lobe, having a compressive effect upon the second-order airway branches to the right lower lobe; B) presence of bilateral hilar lymphadenopathies (red arrows)

A 70-year-old male with a history of coronary artery disease and gastroesophageal reflux disease (GERD) presented to the Scleroderma Clinic. Although he did not have a previous history of scleroderma, he presented with an acute onset of Raynaud’s phenomenon, followed by skin thickening involving his hands and thighs. He reported a 10-pound weight loss over the last several months attributed to poor appetite. Subsequently, the patient experienced muscle weakness, difficulty climbing stairs, and exertional dyspnea. A physical examination showed skin thickening of fingers bilaterally with involvement of the face, chest, arms, and thighs, as well as a tendon friction rub, consistent with diffuse scleroderma. Strength was graded as 5/5 upper extremities, 4/5 thighs, and 4/5 distal lower limbs. Laboratory analyses revealed a positive neutralizing antibody (NAB), an antinuclear antibody (ANA) with HEp-2 substrate titer of 1:320 (NR: < 1:160) in a speckled pattern, scleroderma antibody (Scl-70) of 3 U (negative < 1 U), centromere antibodies 2 U (negative < 1 U), an erythrocyte sedimentation rate (ESR) of 15 mm/hr (NR: 0 - 20 mm/hr), a C-reactive protein (CRP) of 0.56 mg/dL (normal value: < 1 mg/dL), a creatine phosphokinase (CPK) of 82 U/L (NR: 26 - 192 U/L), and an aldolase of 11 U/L (NR: 1 - 7.5 U/L). His creatinine was within NR, and the rheumatoid factor (RF), ENA 11, and anti-double-stranded deoxyribonucleic acid (anti-dsDNA) antibodies were negative. Electromyography (EMG) showed axonal polyneuropathy with active degeneration distally and mild lumbosacral radiculopathy of the L4, L5, and S1 nerve roots. A chest x-ray (CXR) showed emphysema, along with mild mediastinal and right hilar lymphadenopathy. Accordingly, the chest computed tomography angiography (CTA) showed a large right hilar mass in association with consolidation to the medial right lower lobe and the presence of bilateral hilar lymphadenopathies (Figure 2). 

Furthermore, a positron emission tomography (PET) scan revealed a mass in the mediastinum, along with a right hilar mass, and supraclavicular and right paratracheal lymph nodes, suspicious of an aggressive neoplasm. Fiberoptic bronchoscopy with needle aspiration revealed a poorly differentiated non-small cell lung cancer, and the patient was referred to an oncologist for further follow-up.

Case #3: paraneoplastic Raynaud’s syndrome

**Figure 3:**
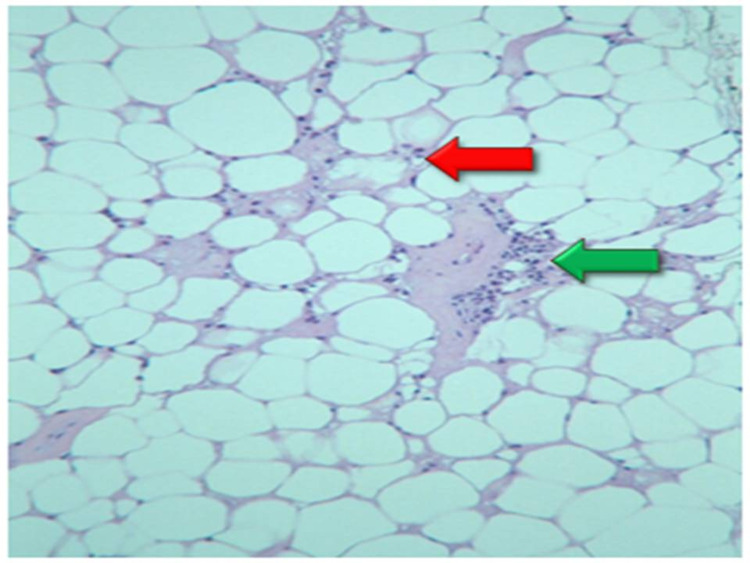
Fat tissue biopsy demonstrated lupus profundus with lobular panniculitis: the hyaline degeneration of fat (red arrow) with lymphocyte infiltration (green arrow)

A 53-year-old male with a history of tobacco use and Raynaud’s phenomenon complained of diffuse arthralgias for the past four to five years. A right knee arthrocentesis demonstrated urate crystals, and the patient was started on allopurinol. Subsequently, the patient developed a necrotic erythematous rash on the right lower extremity, and tissue biopsy demonstrated lupus profundus with lobular panniculitis (Figure 3).

Laboratory results were significant for leukopenia of 2.1 × 109/L (NR: 4.5 - 11.0 × 109/L) and an absolute neutrophil count (ANC) of 1,100/mm^3^ (NR: 1,500 to 8,000/mm^3^) with a low complement 4 (C4) level. Serological workup revealed a positive anti-dsDNA antibody of 161 IU/ml (NR: < 30 IU/ml) and a negative ANA on immune fixation (IFE), and ENA 11. A CT scan of the chest, abdomen, and pelvis showed a 6 mm nodule in the left upper lobe of the lung. The patient was diagnosed with undifferentiated connective tissue disease, and he was started on prednisone, 10 mg, and hydroxychloroquine, 200 mg daily. The patient was referred to the pulmonology service to follow up on the nodule at six months and one year; the nodule has remained stable in size. The patient was recommended to undergo upper endoscopy and colonoscopy, which he declined. Later, the patient developed paresthesias of his feet, and an EMG showed peripheral motor axonal polyneuropathy. A sural nerve biopsy revealed small leukocytoclastic vessel vasculitis. Therefore, the patient was restarted on 60 mg of prednisone daily. The patient did not tolerate mycophenolic acid or azathioprine due to gastrointestinal symptoms, and he was started on monthly intravenous cyclophosphamide for 12 months. Subsequently, the skin rash and polyneuropathy improved. On follow-up, three years after the initial diagnosis, abdominal and pelvic MRIs reported bilateral avascular necrosis and abnormal osseous structures concerning bone metastasis. The pan-CT scan showed metastatic disease, and the bone biopsy in the right poster iliac crest showed squamous cell carcinoma.

Case #4: paraneoplastic inflammatory myopathy

**Figure 4:**
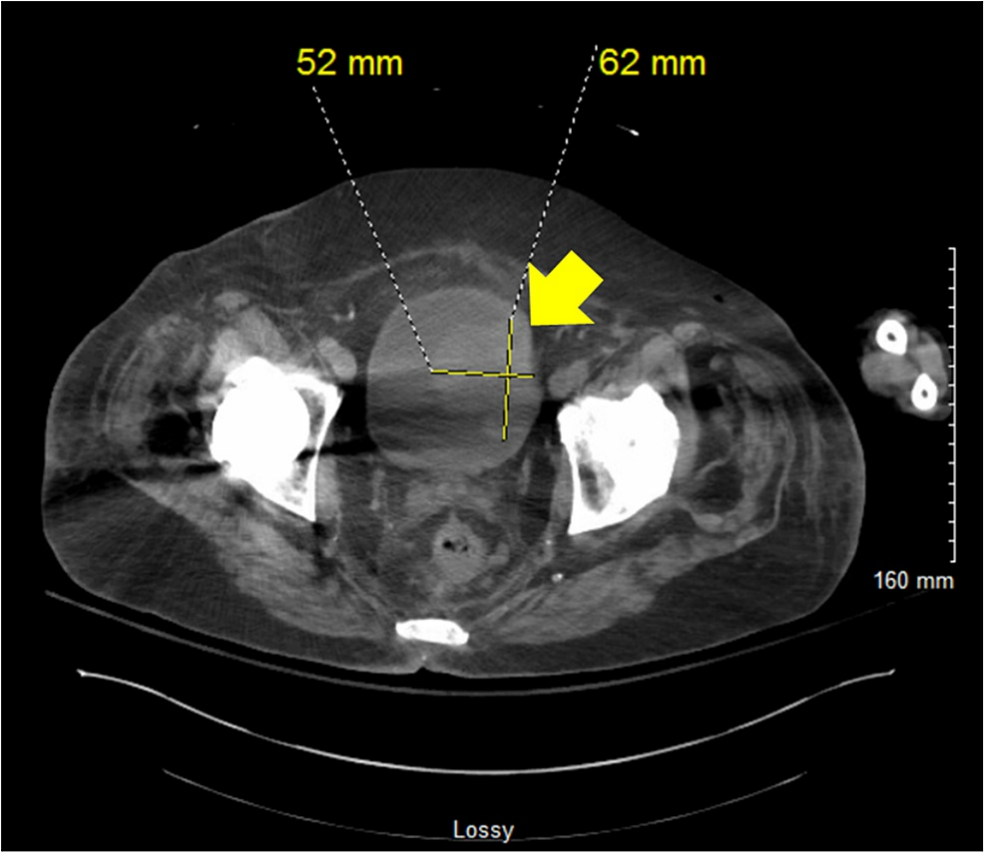
Computed tomography (CT) of the abdomen and pelvis without contrast showed a lobular mass along the left side wall of the urinary bladder measuring 5.2 cm (yellow arrow) with irregular thickening, raising concern for a neoplastic process

A 72-year-old male developed bilateral leg pain after a fall. An x-ray was negative for fracture, and a Doppler ultrasound (US) was negative for deep venous thrombosis (DVT). Subsequently, the patient developed progressive weakness with difficulty getting in and out of his car and climbing stairs. The patient reported decreased appetite, progressive fatigue, pain in his shoulders and calves, and swelling of his lower limbs. He was also found to have bluish discoloration of his fingers and toes on exposure to cold. Laboratory investigation revealed hyponatremia of 124 mEq/dL (NR: 135 - 145 mEq/dL), and he was subsequently admitted to a local hospital. A CT of the abdomen and pelvis revealed mesenteric panniculitis, as well as enlarged lymph nodes in the axillary, periportal, and peripancreatic regions. Other significant findings included an elevated thyroid-stimulating hormone (TSH) level and persistent leukocytosis with a negative pan culture. The patient was transferred to an academic hospital with digital ischemia and painful fingers. The left hand showed purplish discoloration of the fingers with ulcers at the tip of the second and third digits. The patient complained of the tenderness of the second forefinger, and a CTA reported patent arterial inflow circulation into both upper extremities. An abdominal CT revealed a bladder wall-based mass with irregular thickening, concerning for neoplasia (Figure 4). It also showed multiple borderline mesenteric lymph nodes with surrounding inflammatory change.

Further laboratory investigation revealed a creatinine kinase (CK) of 1,082 (NR: 55 - 170 U/L), a beta 2 glycoprotein 1 antibody (B2GP1) IgA of 41 U/ml (negative < 20 U/ml), a positive NAB, an anti-histidyl-tRNA-synthetase (anti-Jo1) of 2 AI (negative < 1 AI), a negative RF, and a borderline low C3 of 80 mg/dL (NR: 80 - 160 mg/dL). Cryoglobulins were negative, as well as ANCA, MPO, and PR3. A repeat TSH level showed a normal value without change on the CK. A muscle biopsy reported necrotic, regenerating fibers and a predominately endomysial inflammatory reaction that indicated an inflammatory myopathy with mild auto-aggressive features. Of note, the patient did not take any medication implicated in causing myositis. A bladder cystoscopy washing reported urothelial cell carcinoma, and tissue pathology showed invasive papillary urothelial carcinoma.

## Discussion

Paraneoplastic RD may present before, concomitant with, or after the diagnosis of cancer. As illustrated by our four cases, it may be difficult to differentiate paraneoplastic cancer syndromes from RD. Nevertheless, it is imperative to recognize malignancy since its treatment almost always results in the improvement of RD. According to Fam et al., paraneoplastic RD can be classified into five broad groups (articular, muscular, cutaneous, vascular, and miscellaneous) outlined in Table 1 [4]. In addition, some general principles that suggest paraneoplastic RD are 1) history of prior malignancy, exposure to carcinogens, or a family history of cancer, 2) late age of onset of > 50 years, 3) prominent constitutional symptoms, such as fever, weight loss, and/or malaise, 4) rapid onset of unusual inflammatory arthritis, 5) close temporal relationship between the onset of paraneoplastic symptoms and the discovery of cancer, 6) absence of metastases to the bones and joints, 7) negative RF, culture results, and synovial fluid crystal analysis, 8) poor response to conventional medical therapy, 9) improvement of symptoms with treatment of the underlying malignancy, and 10) reappearance of the paraneoplastic symptoms with tumor recurrence.

**Table 1:** Classification of Paraneoplastic Rheumatic Disorder into Five Groups: Articular, Muscular, Cutaneous, Vascular, and Miscellaneous

Classification of Paraneoplastic Rheumatic Disorder into Five Groups				
Articular	Muscular	Cutaneous	Vascular	Miscellaneous
Hypertrophic osteoarthropathy (HOA); carcinoma polyarthritis; amyloidal arthritis; secondary gout; relapsing polychondritis; benign edematous polysynovitis/remitting seronegative symmetrical synovitis with pitting edema (RS3PE); adult-onset Still’s disease (AOSD); sacroiliitis	Dermatomyositis (DM); polymyositis (PM); necrotizing myopathy (NM); inclusion body myositis (IBM); Lambert-Eaton myasthenic syndrome (LEMS); myasthenic syndrome (MS)	Palmar fasciitis and arthritis; panniculitis and arthritis; erythema nodosum; eosinophilic fasciitis; scleroderma-like syndromes; osteosclerotic myeloma/polyneuropathy, organomegaly, endocrinopathy, monoclonal protein, skin changes syndrome (POEMS syndrome); granulomatosis with polyangiitis	Paraneoplastic vasculitis; granulomatosis with polyangiitis; Raynaud’s syndrome and digital gangrene; erythromelalgia	Reflex sympathetic dystrophy syndrome; Jaccoud arthropathy (JA); lupus-like syndrome; relapsing polychondritis; multicentric reticulohistiocytosis; pyogenic arthritis; oncogenic osteomalacia

Paraneoplastic rheumatologic syndromes

Paraneoplastic Cutaneous Small-Vessel Vasculitis (CSVV) and C3 Glomerulopathy

Cutaneous small-vessel vasculitis (CSVV) is the most common paraneoplastic vasculitis syndrome [5]. Similar to other vasculitides, CSVV is most commonly associated with hematologic malignancies, such as chronic myelomonocytic leukemia, non-Hodgkin's lymphoma, Hodgkin's disease, and chronic lymphocytic leukemia [6-7]. Among solid cancers, lung (non-small-cell), prostate, colon, breast, and kidney cancers are most frequently described concurrently with CSVV. Concomitant ovarian cancer, hepatocellular carcinoma, pancreatic cancer, and pheochromocytoma occur less commonly [8-11]. The typical histopathological finding of paraneoplastic CSVV is leukocytoclastic vasculitis, consisting of a small vessel, immune complex-mediated vasculitis with neutrophil infiltration of the dermal capillaries and venules. Fibrinoid necrosis of the vasculature can occur, and red blood cells can escape from the vessels and present in the dermis as palpable purpura [12]. Paraneoplastic CSVV is most commonly reported as palpable purpura in the legs, along with constitutional symptoms (fever, weight loss, and diaphoresis) and cytopenias [13]. In a retrospective study at Mayo Clinic, 17 patients who had been diagnosed with CSVV associated with solid organ malignancy presented with palpable purpura, two with urticarial lesions, four with arthritis, and one with interstitial pulmonary fibrosis [7]. The most common solid cancer was lung (n = 4), followed by breast (n = 3), prostate (n = 2), renal (n = 2), thyroid (n = 1), bladder (n = 1), gallbladder (n = 1), and peritoneal (n = 1) cancers. The mechanism behind paraneoplastic CSVV is unclear, but a possible hypothesis is that the tumor releases neoantigens, resulting in immune complexes that deposit in small vessels. Afterward, neutrophils are activated and complement fixation occurs, resulting in the release of oxygen radicals, lytic enzymes, and inflammatory mediators [6-7, 14]. Most case reports and series report that patients with paraneoplastic CSVV are older than those with CSVV unrelated to malignancy and that the duration of paraneoplastic CSVV is an average of 14.19 + 4.52 days, compared to 12.32 + 5.42 days in CSVV that is unrelated to malignancy (p = 0.03). In addition, these reports demonstrate that paraneoplastic CSVV is not associated with a drug side effect and responds better to cancer treatment [15-17]. Our case #1 described a paraneoplastic CSVV secondary to a pancreatic tumor that initially presented with a diffuse rash and subsequently manifested as a leg rash refractory to treatment. Contrary to Podjasek et al., our case #1 of cutaneous paraneoplastic RD did not respond to topical steroid treatment. CSVVs associated with solid tumors may be more resistant to standard treatment than those associated with hematologic tumors [7].

Another interesting point in our case #1 is that the patient presented with acute kidney injury with hematuria. A subsequent kidney biopsy showed the presence of C3 complement with the absence of antibodies; this increases the suspicion for C3 glomerulopathy [18]. C3 glomerulopathy is a rare and complex renal disease caused by complement dysregulation [18-19]. The typical presentations are proteinuria, hematuria, and hypertension. The main laboratory finding is a low complement level, specifically C3 [20]. A renal biopsy will show glomerular C3 staining of at least two orders of magnitude greater intensity than for any other immune reactant. Electron microscopy may show two subtypes of C3 complement distribution: dense deposit disease (DDD) characterized by the presence of deposits within the glomerular basement membrane (GBM) and C3 glomerulonephritis (C3GN), where the deposit is located in the mesangium and along the subendothelial side of the GMB. The main pathophysiology of C3 glomerulopathy is the dysregulation of the alternative pathway which occurs in the fluid phase. As we mentioned above, the alternative pathway is constitutively active by spontaneous hydrolysis of the reactive thioester on C3 [21]. This leads to the formation of two C3 convertases, via the classical pathway by C4b2a or the lectin pathway by C3bBb. The end product is the C5a (a potent anaphylatoxin) and C5b which will generate the soluble C5-9 or the membrane attack complex (MAC) that induces cell lysis. Multiple factors can contribute to the activation of the alternative pathway, such as autoantibodies, nephritic factors (NeFs) that stabilize the C3 and C5 convertases increasing their half-life and consumption, and genetic abnormalities [22]. Common genetic abnormalities affect the C3 gene, complement factor B (CFB), complement factor H (CFH), complement factor I (CFI), and complement factor H-related (CFHR). Most of the mutations in C3 will alter the recognition sites for the binding of factor H (FH) and factor I (FI) whose main function is the inhibition of the activation of C3 convertase [20]. This overactivation of the fluid phase will lead to C3b deposition on the glycocalyx of GBM and activation of the formation of C3 convertase and C5 convertase with subsequent cellular lysis. There is a possible association between C3 glomerulopathy and malignancy, such as monoclonal gammopathies [23]. Our patient presented with solid cancer, and unfortunately, we did not perform electron microscopy. Thus, it is unclear whether or not this was a true C3 glomerulopathy and if there was any association with the solid tumor.

In addition, our patient presented with elevated serum IgG4 levels. This presented a diagnostic dilemma that raised concern for autoimmune pancreatitis secondary to IgG4-related disease (IgG4-RD) autoimmune pancreatitis. However, the presence of elevated IgG4 does not always correlate with autoimmune pancreatitis [24]. The distinction between autoimmune diseases versus cancer is very challenging. Autoimmune pancreatitis can present with lymphoplasmacytic cell infiltration and fibrosis and create the appearance of a mass in the pancreas in radiological tests [25]. A corticosteroid trial and/or biopsy can establish a definitive diagnosis; in our case, a biopsy showed definitive intraductal adenocarcinoma [26-27].

Scleroderma is a Paraneoplastic Syndrome

Scleroderma, or systemic sclerosis (SSc), is described as skin fibrosis due to a combination of immunological dysregulation, vasculopathy, and hyperproduction of extracellular matrix by fibroblasts. Usually, it affects women between their 30s and 50s [28]. A recent clinical trial confirmed that SSc was associated with an increased incidence of cancer [29]. The most commonly associated cancers were esophageal due to Barrett’s esophagus, lung due to interstitial lung disease (ILD), vaginal and vulvar, multiple myeloma (MM), non-Hodgkin's lymphoma, stomach, and breast [30]. However, Shah et al. suggested SSc as a paraneoplastic disease [29]. Four pathogenic situations might occur SSc initiation independent of the tumor, the induction of SSc by the tumor, the induction of the tumor by SSc, or tumor induction by immunosuppressive treatment. Therefore, the identification of a progenitor in each case may be very challenging and the management is significantly different. A short time between SSc and a cancer diagnosis may suggest a paraneoplastic disease. Retrospective studies with a total of 6,641 scleroderma patients showed a strong correlation between anti-ribonucleic acid (RNA) polymerase III antibody positivity and the rapid onset of the tumor [29, 31-32]. The possible biologic explanation is that tumor cells spread the RNA polymerase (Pol) III mutated gene, and T-cells recognize the antigen and promote the production of B cell-specific antibodies for tumor antigens with RNA mutations. Finally, cross-reactivity with self-antigens may occur, initiating SSc. Patients with paraneoplastic SSc-like symptoms are most commonly older. Marek et al. described possible characteristics that suggest paraneoplastic SSc rather than the primary disease: a short time interval between the diagnosis of cancer and SSc, older age, a family history of cancer, a history of exposure to carcinogens, asymmetric skin fibrosis, asymmetric Raynaud’s phenomenon, lack of abnormalities typical for SSc in capillaroscopy, lack of antibodies characteristic of SSc, general symptoms (such as fever and weight loss), lack response to standard treatment, the disappearance of SSc symptoms after cancer treatment, and their reappearance when cancer reactivates [33]. Lung cancer, breast cancer, and T-cell lymphoma may present with SSc two to three years prior to the diagnosis of cancer [34]. Our case #2 with cutaneous paraneoplastic RD had high suspicion of paraneoplastic RD due to his early presentation. A patient with SSc and an atypical presentation of polyneuropathy who was diagnosed with lung cancer within two months of symptom onset suggests paraneoplastic SSc.

Paraneoplastic Raynaud’s Phenomenon and Possible Lupus-like Syndrome

Raynaud's phenomenon (RP) is a relatively common clinical disorder with an overall prevalence of 1% - 8% in the general population [35]. RP is a transient, acral, vasospastic phenomenon that manifests with characteristic color changes. This vasospasm classically is triggered by cold temperature and/or shifts in temperature, climate, or emotional state. RP is usually classified as primary, without an underlying disorder, in 87% of cases and the remaining cases are classified as secondary, in association with another disorder, such as autoimmune disease, cancer, smoking, or certain medications [36]. This phenomenon is highly localized and affects the arterial inflow of specific skin areas, such as fingers, toes, the tips of the nose, and ears. These sites are distinct from other skin areas; they have a high density of arteriovenous anastomoses, bypass capillaries, and direct connections between arterioles and venules. The main function of the arteriovenous anastomoses is thermoregulation and nutritional support to the skin [37]. Therefore, cold-induced cutaneous vasoconstriction is mediated by a reflex increase in sympathetic constrictor nerve activity and local cold-induced amplification of the sympathetic response. In persons with RP, the already-heightened sympathetic response due to an increase in alpha-2 adrenergic sensitivity produces vasoconstriction in these specialized areas that are further amplified in intensity and scope [37]. Exposure to cold can evoke intense sympathetic-mediated vasoconstriction throughout this vascular network, including upstream arteries, which undergo vasospasm. In cold temperatures, the sympathetic nervous system causes the release of vasoconstricting neuropeptides and norepinephrine, leading to arteriole vasoconstriction and reduced blood flow. In secondary RP, the underlying disorder is responsible for the disruption of normal vessel reactivity to cold temperatures. The endothelial function of the vessels themselves tends to be compromised, leading to vasoconstriction and disrupted blood flow. In addition, endothelin-1 is released by endothelial cells, leading to vasoconstriction. The third group, although not very well-described, is RP as a paraneoplastic disease. It has been reported in association with sarcomas, lymphomas, leukemias, and ovarian cancer. The pathophysiology is not well understood but may be similar to that previously described. Some of the paraneoplastic RP characteristics are similar to the characteristic presentation of secondary RP: unusual episodes, asymmetrical distribution, pain in nature, ischemic complications, and occur in those more than 50 years of age [38]. It can present in any stage of cancer and may be associated with disease activity, given the improvement of symptoms that may be seen after successfully treating underlying cancer [39]. In our case #3 of vascular and undifferentiated paraneoplastic RD, the patient had a history of stable RP with subsequent development of a necrotic erythematous rash, lobular panniculitis, vasculitic neuropathy, and positive anti-dsDNA antibodies without ANA antibodies. This constellation of findings suggested a lupus-like syndrome. The lupus-like syndrome is a rare entity that can mimic clinical, histological, and immunological features of SLE and can be associated with malignancy and/or certain medications [2-3, 40]. Although the underlying pathophysiology is unknown, there is a debate if dysregulation of the immune system is induced by neoplasia or if the immune reaction against tumor-derived antigen promotes the formation of auto-antibodies [41]. Presentation of the lupus-like syndrome can precede the diagnosis of underlying malignancy by at least five years, making the recognition of this paraneoplastic condition a challenging and difficult clinical situation [42-43]. The lupus-like syndrome was frequently associated with hematologic malignancy, such as hairy cell leukemia, non-Hodgkin’s lymphoma, and solid tumors (like breast carcinoma, ovarian carcinoma, and lung cancer) [44-47]. Skin lesions were reported as initial manifestations of paraneoplastic RD with a positive response to steroids. Our patient showed further improvement after a course of a high dose of prednisone. However, the definitive management is to treat underlying cancer. A possible dilemma, in this case, is whether the squamous cell carcinoma triggered the patient’s paraneoplastic RD and autoimmune condition or if the patient had an independent autoimmune disorder.

Cancer-associated Myopathy Paraneoplastic Disorder

A recent review defines cancer-associated myopathy (CAM) as myopathy that occurs two years before or three years after the diagnosis of cancer [48]. There are four possible forms of myositis described: dermatomyositis (DM), polymyositis (PM), necrotizing myopathy (NM), and inclusion body myositis (IBM). In a retrospective study, DM and PM were two of the more common presentations - the incidence of DM as a CAM was 20.5% (30 out of 146 patients) while the incidence of PM was 4.3% (13 out of 304 patients) [49]. The histopathology of DM is predominantly inflammatory infiltration at perivascular sites or within the interfascicular septae. The main pathological characteristic is the distribution of atrophic, degenerating, and regenerating fibers at the periphery of the fascicle, as well as perifascicular atrophy involving both type 1 and type 2 muscle fibers which may affect two to 10 layers [50]. On the other hand, PM is characterized by inflammatory mononuclear cell invasion surrounding the sarcolemma of the muscle fibers. Meanwhile, NM is a rare entity characterized by massive necrosis of the muscle fibers in the absence of inflammation. Paraneoplastic NM is associated with autoimmunity and characterized by rapid, symmetrical, proximal muscle weakness with acute or subacute onset of dysphagia. The biopsy is characterized by randomly distributed necrotic muscle, along with fibers in different stages of regeneration, but mononuclear cell infiltration is sparse or absent. This minimal infiltration helps to distinguish NM from other CAM [51]. Finally, a cohort study showed that 23% of patients with IBM (12 out of 52) were diagnosed with malignancy [52]. IBM commonly presents as an insidious, painless, progressive onset of proximal and distal muscle weakness that occurs predominantly in males greater than 50 years old and usually is resistant to steroid treatment [53]. IBM is similar to PM in terms of inflammatory cell findings. However, the presence of one or more irregular and variably-sized vacuoles, characterized by basophilic granular deposits around the edges (rimmed vacuoles), eosinophilic cytoplasmic inclusions, and single or multiple amyloid intracellular deposits identified with Congo red staining, is specific for IBM. Different malignancies have been reported with IBM, such as chronic lymphocytic leukemia (CLL), endometrial carcinoma, bladder carcinoma, and hepatocellular carcinoma [54-55]. The average age of IBM onset is 56.6 years with a male sex odds ratio (OR) of 1.92 [56]. The most common manifestations are skin rashes and frequent falls due to asymmetric, slowly progressive quadriceps and hip flexor muscle weakness. Patients can also present with finger flexor weakness sparing thenar, hypothenar, and extensor muscles. Some of the common extramuscular manifestations are arthralgia, dysphagia, and heart and lung involvement [57]. The presence of ILD, arthritis, arthralgia, or anti-Jo-1 antibodies was associated with a lower risk of malignancy. In 15% of cases, IBM is associated with autoimmune disorders, such as systemic lupus erythematosus (SLE), Sjorgren’s syndrome, thrombocytopenia, or sarcoidosis [58].

CAM is considered a paraneoplastic disorder due to an increased incidence of cancer during the first three years of myositis, its resistance to standard treatment, its response to treatment of the underlying malignancy, and its recurrence as the cancer relapses. CAM should be suspected in older patients, those with features of dermatomyositis, those with an absence of interstitial lung disease, those with findings of severe cutaneous necrotizing inflammation on biopsy, and those who poorly respond to myositis treatment [59]. Laboratory workup may reveal elevated muscle enzymes, such as creatinine kinase and aldolase. The detection of autoantibodies can assist risk disease stratification, and these antibodies may be either myositis-specific or myositis-associated. Myositis-specific antibodies are anti-Jo1, anti-signal recognition particle (anti-SRP), anti-Mi-2, anti-MJ, and anti-p155. Myositis-associated antibodies, which are commonly found in other rheumatological diseases, such as Sjogren’s syndrome, mixed connective tissue disease, and scleroderma, are anti-Ro, anti-La, anti-ribonucleoprotein (anti-RNP), anti-SM, and anti-Scl. The anti-p155 is a myositis-specific antibody that was recently discovered and is reactive against the transcription intermediary factor-1 gamma (TIF1-ɣ) protein involved in cell proliferation, immunity, and carcinogenesis. Anti-p155 is highly associated with CAM and its presence is associated with a 27-fold increased chance of developing cancer compared with its absence [60]. CAM is resistant to standard treatment but usually improves after treating the underlying neoplasia; additionally, recurrence of cancer has been associated with relapse of CAM [61]. Due to the high incidence of cancer during the first three years of myositis, annual cancer surveillance must be emphasized for the first three to four years [48, 62]. Recommended surveillance is a complete pelvic/prostate examination, chest radiography, and age and gender-appropriate cancer screening. 

Our patient (case #4) was an elderly male with ongoing Raynaud's phenomena, who presented with proximal muscle weakness and was found to have elevated muscle enzymes, positive anti-Jo1 antibodies, and biopsy findings of endomysial infiltration characteristic of PM. A repeat TSH level showed a normal value with a high CK level, and metabolic myopathy was ruled out. Due to this atypical presentation of PM in an elderly male, malignancy was suspected and diagnosed. This case of paraneoplastic RD highlights the importance of maintaining a high suspicion of CAM in patients with atypical presentations of necrotizing myositis.

Paraneoplastic Vasculitis

Paraneoplastic vasculitis is infrequent. According to Fain al., paraneoplastic vasculitis represents less than 5% of all vasculitides [5]. Hematological cancer is more frequently reported concurrently with paraneoplastic vasculitis than solid malignancies, and the vasculitis may present one month before or after malignancy recognition. In a prospective study by Solans-Laque, et al., 15 patients diagnosed with vasculitis and a solid tumor were followed for 12 months; the diagnosis was cutaneous leukocytoclastic vasculitis in 60%, giant cell arteritis in 20%, Henoch-Shonlein purpura in 13%, and polyarteritis nodosa in 7% of the patients [11]. The most common cancers associated with vasculitis were urinary cancer (40%), lung cancer (26.7%), and gastrointestinal tract cancer (26.7%). Emmi et al. reported an increased incidence of thrombosis in the presence of Behcet's syndrome, ANCA-associated vasculitis, and giant cell arteritis flares [63]. Patients with paraneoplastic vasculitis may present with typical vasculitis signs and symptoms, along with severe weight loss and arthritis that do not respond to typical therapies, such as glucocorticoids or immunosuppressive agents. However, treatment of the underlying malignancy may result in the resolution of the vasculitis.

Paraneoplastic Arthritis

Paraneoplastic arthritis (PA) is not well-described, but case series report its manifestations as joint pain, swelling, and the presence of synovitis on examination [64]. The presentation is similar to rheumatoid arthritis, and it may manifest as the initial presentation of malignancy. According to Manger et al., PA predominantly affects males in a ratio of 1.7:1, with a median age of 54 + 5 years. The most common cancers associated with PA were non-small cell lung cancer, breast cancer, lymphomas, leukemias, and myelodysplastic syndromes [65-66]. Although the mechanism is not well established, potential pathophysiologic mechanisms have been proposed. There may be a direct invasion of the synovium, soft tissues, or bone by the primary or secondary tumor, leading to PA. In addition, immunologic cross-reactivity between the tumor and synovium was suggested by synovial infiltration of tumor-specific T-cells in a patient with renal cell carcinoma and PA [67-68]. Various studies describe that arthritis can manifest 10 months before malignancy, but the conditions can also present together. The common characteristic presentation of PA is associated with advanced age, acute onset of symptoms, an asymmetric pattern of joint involvement (predominantly, the lower extremities), and disproportionate pain compared to the physical examination findings [68]. Most cases of PA have been described as seronegative arthritis [69]. However, published reviews have described that 27.2% of patients had positive RF, 19% had ANA, and 10.7% had anti-CCP anti-cyclic citrullinated peptide (anti-CCP) antibodies [70-72]. Contrary to most patients with inflammatory arthritis, patients with PA usually have a poor response to corticosteroids and disease-modifying antirheumatic drugs (DMARDs). In most cases, treatment of the underlying malignancy results in regression of the symptoms.

Immune Check Inhibitor-Related RDs

The immune check inhibitor (ICI) is a new cutting-edge treatment for most oncological diseases. With ICIs, newly described immune-related adverse events (irAEs) are reported and might present as RDs [73]. The mechanism behind ICIs is by activating the T-cells through the inhibition of cytotoxic T-lymphocyte-associated antigen-4 (CTLA-4) or programmed cell death-protein 1/programmed death-ligand 1 (PD-1/PD-L1) to enhance the immune system against the tumor cells. ICIs dysregulate the immune system and may subsequently result in manifestations, such as arthralgia/arthritis, myalgias/myositis, polymyalgia rheumatica (PMR), giant cell arteritis (GCA), lupus, rheumatoid arthritis (RA), and Sjogren's syndrome [74-76]. Also, more cases report worsening in established RD after receiving ICI therapy [77]. According to Kostine et al., myalgias and arthralgias were the most common reported irAEs [73]. The most common joint complications were located in the shoulder, metacarpophalangeal (MCP) joints, and proximal interphalangeal (PIP) joints of the hands (incidence of 50%), followed by the knees and wrists (40%). Some studies report that two-thirds of patients demonstrate an elevated CRP with synovial fluid analysis demonstrating an inflammatory reaction with polymorphonuclear predominance [78-81]. Some cases report seronegative disease with atypical features, such as PMR with negative inflammatory markers or seronegative RA-like symptoms [74, 78]. Regarding myositis, symptoms onset is characteristic of acute or subacute myalgia (38%), proximal muscle weakness (50%), ptosis/diplopia (25%), and dysphagia/dysarthria (25%) [82]. The patient may also manifest with life-threatening dyspnea and concurrent myocarditis. Therefore, cardiac evaluation, such as troponin, electrocardiography (EKG), and echocardiogram, is necessary if suspected of myocarditis. Patients may also present with myasthenia gravis, which should be part of the differential. Most patients will report high CPK levels with an average of 2,500 UI/L. Myositis-specific antibodies are reported negative in most cases.

Sicca syndrome, including xerostomia (80%), xerophthalmia (60%), and arthralgia (10%), is reported in several case reports [74-75, 77-78, 83-85]. A recent extensive case series (20 patients) reported T-cell infiltration, mainly composed of a cluster of differentiation (CD)3+ T-cells with the predominance of CD4+ over CD8+ T-cells [86]. This is different from the classical Sjogren’s syndrome characterized by CD20+ cells and variable germinal center formation [69, 87].

Separate case reports described GCA with the use of pembrolizumab and nivolumab [86, 88]. The patient may manifest characteristic signs and symptoms of GCA, such as loss of vision, severe or unusual headache, temporal tenderness, temporal vascular prominence, and jaw claudication. However, the patient may present with negative inflammatory markers. Some patients manifest concurrent PMR; however, most of the cases reported GCA alone [89]. Biopsy shows no differences from the non-ICI GCA. Overall, rheumatologists need to work along with oncologists in a patient receiving ICI to diagnose and treat possible irAE. Treatment consists of non-steroid anti-inflammatory drugs, steroids, and in some cases, DMARDs.

## Conclusions

Paraneoplastic RD is challenging with atypical presentations that require a thorough clinical examination. For that reason, the rheumatologist must be an expert diagnostician and be cognizant of atypical presentations of paraneoplastic RD to correctly manage both the underlying malignancy and RD diseases. As a result, more case reports and reviews are necessary to raise awareness and inform both rheumatologists and oncologists of such cases. Moreover, the management of RD and cancer requires oncologists and rheumatologists to work collaboratively. Furthermore, the management of RD and cancer requires oncologists and rheumatologists to work collaboratively. Modern therapy, such as antineoplastic, ICI, and immunosuppressive treatment, can induce RDs and cancers. Therefore, comprehensive, interdisciplinary, timely, and proper management of suspected paraneoplastic RD is of utmost importance.
